# REal-world treatment outcomes after delayed intRavitreal therapy in center-involving diabetic macular edema – RETORT study

**DOI:** 10.1186/s40942-023-00463-y

**Published:** 2023-03-30

**Authors:** Sai Prashanti Chitturi, Ramesh Venkatesh, Rubble Mangla, Yash Parmar, Rohini Sangoram, Naresh Kumar Yadav, Jay Chhablani

**Affiliations:** 1 Dept. of Retina and Vitreous, Narayana Nethralaya, #121/C, 1st R Block, Chord Road, Rajaji Nagar, 560010 Bengaluru, Karnataka India; 2 Medical Retina and Vitreoretinal Surgery, University of Pittsburgh School of Medicine, 203 Lothrop Street, Suite 800, Pittsburg, PA 15213 USA

**Keywords:** Diabetic macular edema, Early treatment, Delayed treatment, Compliance, Outcomes

## Abstract

**Purpose:**

To compare real-life data on delayed intravitreal treatment of diabetic macular edema (DME) patients to early treatment.

**Methods:**

In this single-centre, retrospective, interventional, comparative study, DME patients were divided into two groups based on when they received treatment: Group 1 - received treatment within 24 weeks and Group 2 - at or after 24 weeks from the time of treatment advice. Visual acuity and central subfield thickness (CSFT) changes were compared at various time points. Reasons for delaying treatment were noted.

**Results:**

The study included 109 (Group 1–94; Group 2–15) eyes. When treatment was advised, demographic profile, diabetes duration, glucose control and VA between two groups were comparable. At this point, CSFT was higher in Group 1 than in Group 2 (p = 0.036). At injection time, Group 2 had better VA and lower CSFT than Group 1 (p < 0.05). Group 2’s VA (53.4 ± 12.67) was significantly lower than Group 1’s (57.38 ± 20.01) after 1-year treatment. At 1-year, CSFT decreased in Group 1 and increased in Group 2. Group 1 had mean improvement of + 7.6 letters and Group 2 had a decline of -6.9 letters. Group 2 required more intravitreal anti-VEGF (median – 3; IQR: 2–4), steroid injections (median – 4; IQR: 2–4) and focal laser sessions (median – 4; IQR: 2–4).

**Conclusion:**

Late-treated DME eyes needed more injections and focal laser sessions than early treated eyes. Adherence to early treatment of DME in real-life will help prevent long-term vision loss.

## Introduction

Diabetic retinopathy (DR) is the leading cause of blindness in the world, and diabetic macular edema (DME) plays a major role in vision loss [1, 2]. All patients with DR are at a risk of developing DME. DME usually appears gradually and causes mild to moderate vision loss [3, 4]. DME develops as a result of prolonged hyperglycaemia, which damages the retinal endothelial cell tight junctions and promotes fluid leakage from retinal capillaries, resulting in the accumulation of subretinal and intraretinal fluid and macular edema [5]. A number of pathogenetic pathways related to inflammation and vascular endothelial growth factor (VEGF) have been identified in the development of DME [6]. Thus, in the current era, intravitreal anti-VEGF and steroid therapy are the leading treatment options for center involving DME (CI-DME).

The presenting visual acuity (VA) has been an important parameter for initiating intravitreal therapy in the management of DME [7]. According to the diabetic retinopathy clinical research network (DRCR.net) protocol V trial, patients with good VA (≥ 20/32) can be observed [8]. According to a number of landmark clinical trials published by the DRCR.net writing group, patients presenting with vision loss (< 20/32), as soon as the DME is diagnosed, early treatment with intravitreal anti-VEGF agents is recommended [9, 10]. If left untreated, DME can cause chronic edema and irreversible changes in the retina, rendering the patient visually impaired. Studies have shown that if the disease is not treated, 20-30% of DME patients will lose at least three lines of vision within three years [11]. As a result, untreated DME has a poor long-term prognosis, and treatment should begin as soon as a patient is diagnosed.

In most instances, the protocol used in clinical trials conducted in an investigator-controlled clinical setting is incompatible to a real-world patient-controlled clinical setting [12]. In the real world, a number of patients either refuse or postpone treatment with intravitreal agents for DME management. There is little information in the literature about the effects of delayed DME management on VA in a real-world clinical setting. The real-world treatment outcomes of observation and treatment in patients with DME with very good VA were published in the OBTAIN study [13]. The study concluded that the majority of DME patients with very good VA (≥ 20/25) maintained vision at 12 months, regardless of whether the DME was treated or not, suggesting that DME eyes with very good VA be closely monitored and treatment considered when a one-line drop in vision is observed. The study findings were consistent with those published in Protocol V of the DRCR.net study [13]. However, there is a lack of information in the literature about real-world experiences with the timing of intravitreal therapy for patients with CI-DME and moderate visual loss (< 20/30).

With this background, we aimed to compare the treatment outcomes of early (< 24 weeks) and delayed (≥ 24 weeks) treatment groups in patients with treatment naïve CI-DME with a presenting VA worse than 20/30 over a 1-year follow-up after treatment initiation in a real-world scenario.

## Methods

In this single-centre retrospective study, clinical databases were searched for all type 2 diabetes mellitus cases with non-tractional treatment-naive CI-DME who visited the retina clinic of a tertiary eye care hospital between June 2017 and December 2020. The presence of retinal edema/thickening within the 1-mm Early Treatment Diabetic Retinopathy Study (ETDRS) circle seen on the retinal thickness map of a macular volume OCT scans obtained on the Heidelberg Spectralis device was defined as CI-DME. The medical records of these cases were reviewed to determine the presenting VA, the time when the intravitreal injection was recommended, and the time when the patient received the first intravitreal injection. Two groups were identified based on the time it took to receive the intravitreal injection from the time of treatment advice: Group 1 (early): eyes treated within 24 weeks of advice and Group 2 (delayed): eyes treated at or after 24 weeks of advice. Eyes with clinical and OCT details at the time of the injection and one year after treatment began were studied. Thus, only non-tractional CI-DME eyes with clinical and OCT details at the time of treatment advice, injection and one year after starting treatment were considered for the study.

The study excluded all other causes of maculopathies mimicking DME and DME caused by traction from vitreomacular interface abnormalities such as epiretinal membrane, taut posterior hyaloid, and vitreomacular traction syndrome. Patients with glaucoma and visual field loss, retinal or optic nerve lesions, or any other factor (e.g., significant cataract) that could impact the visual outcome were omitted from the study. Patients with insufficient systemic or ocular information were excluded as well. The study excluded eyes that had recently undergone pan retinal photocoagulation within the previous three months. Eyes with poor quality OCT images were not included in the study.

All eligible patients’ medical records were reviewed, and the following information was gathered: age, gender, affected eye, duration of diabetes mellitus, associated medical conditions, VA at presentation, at the time of injection, and one year after starting therapy, severity of DR, and treatment received by the patient. The International Clinical Disease Severity Scale was used to categorize DR severity into mild, moderate and severe non-proliferative DR and proliferative DR [14]. VA was recorded in Snellen units in the study.

The Spectralis, Heidelberg machine provided the OCT images (Heidelberg Engineering, Germany). A 25-line horizontal raster macular volumetric scan centered at the fovea was performed and used for the study, with 512 A-scans per line and a 30° scanning area. The following information was obtained from the OCT images: the presence of CI-DME at presentation and central subfield thickness (CSFT), which was retinal thickness measured in the 1 mm ETDRS circle in an automated manner. The findings in the OCT scans were recorded at the time of initial presentation, just prior to the intravitreal therapy and at 1-year after starting the therapy. Changes in the outer retinal layers, such as external limiting membrane (ELM) and ellipsoid zone (EZ) layer discontinuity, and changes in the retinal pigment epithelium (RPE) layer at the fovea, were given special attention in eyes where VA did not correlate with central retinal thickness and other ocular findings.

The treatments both groups received during the one-year follow-up period following the initiation of intravitreal therapy were documented. During the course of the study, retina specialists treated eyes at their discretion. The choice of intravitreal anti-VEGF (Avastin®, Accentrix®, Razumab®, or Eylea®) and/or steroid (Ozurdex® implant or triamcinolone acetonide) agent, the treatment protocol to be followed, the time gap between two consecutive intravitreal injections, the decision to switch from one treatment modality to another, and the decision to discontinue therapy were entirely at the discretion of the treating clinician. The protocol for repeating intravitreal injections was based on the intravitreal medication administered during the previous treatment session. For intravitreal anti-VEGF agents such as Avastin®, Accentrix®, and Razumab®, injections were repeated after four weeks, whereas injections for Eylea® were repeated after eight weeks. The eyes were retreated with intravitreal Ozurdex® and triamcinolone acetonide after a minimum of 12 and 8 weeks, respectively. On the OCT, intravitreal agents were discontinued when the macular edema resolved completely and the foveal contour returned to normal. In most instances, focal thermal laser therapy was reserved for DME cases that did not involve the foveal center. The focal laser treatment protocol followed was in accordance with the modified ETDRS treatment guidelines, with a burn size of 50 μm, a burn duration of 0.05 to 0.1 s, and a mild grey-white burn treating all areas of diffuse capillary leakage or non-perfusion at a distance of 500 to 300 μm superiorly, nasally, and inferiorly from the centre of the macula and at 500 to 3500 μm temporally from the centre of the macula. There were no burns within 500 μm of the optic disc.

The study’s outcome measures were as follows:

A) The proportion of patients who postponed treatment for ≥ 24 weeks after receiving treatment advice.
B) Differences in VA and CSFT between the two groups at the time of injection and one year after starting therapy.
C) The percentage of patients in both groups who lost 5 ETDRS or more and the reasons for this at the 1-year follow-up visit.
D) Intravitreal treatments received in both groups during the one-year follow-up period.

All data were collected and analysed in accordance with the policies and procedures of the local Institutional Review Board as well as the principles outlined in the Helsinki Declaration.

### Statistical analysis

All data were analysed using GraphPad Prism version 9.4.1 (681) for Windows, GraphPad Software, San Diego, California USA, www.graphpad.com. The Kolmogorov-Smirnov normality test showed the data sets to be of the non-parametric variety and hence only non-parametric statistical tests were used in this study. Snellen’s VA was converted to approx. ETDRS letters using the formula 85 + 50 x log (Snellen fraction) [15]. Quantitative variables between the 2 groups (Group 1 and Group 2) were analysed using the Mann-Whitney U test. Chi-square test was used to compare the categorical data between the 2 groups. The Wilcoxon signed-rank test was used to compare changes in VA, CSFT, and treatments received in the two groups at various points throughout the study. P values < 0.05 were considered statistically significant.

## Results

One hundred and nine eyes of 93 patients who met the inclusion criteria were considered for the study. The average age of the study participants was 65.34 ± 8.90 years and there were 74 males and 19 females in the study. Mean duration of diabetes mellitus of 16.39 ± 7.88 years in the study. The mean HbA1C level was 8.4% and 42% of the patients (n = 39) did not have any associated systemic illness. Table 1 shows the DR severity grading in the study participants. In the study, the mean approximate ETDRS VA was 47.14 ± 17.1 letters and the mean CSFT was 516.0 ± 141.8 μm. Treatment with intravitreal agents was administered within 24 weeks in 94 (86%) of the eyes and after 24 weeks in 15 (14%) of the eyes from the time of treatment advice in the study.

**Table 1:** Demographic, clinical and optical coherence tomography (OCT) findings of the study participants:

Variable	Value
No. of patients (n)	93
No. of eyes (n)	109
Age (years)	65.34 ± 8.90
Males: Females	74:19
Duration of DM (years)	16.39 ± 7.88
HbA1c (%)	8.4 ± 0.974
Coronary artery disease (n, %)	7 (8)
Hypertension (n, %)	49 (53)
Hypercholesterolemia (n, %)	3 (3)
Chronic kidney disease (n, %)	4 (4)
Cerebrovascular accident (n, %)	2 (2)
No systemic illness (n, %)	39 (42)
Moderate NPDR (n, %)	26 (24)
Severe NPDR (n, %)	38 (35)
PDR (n, %)	45 (41)
Visual acuity (ETDRS letters)	47.14 ± 17.1
Central subfield thickness (microns)	516.0 ± 141.8
No. of eyes who received intravitreal injections for DME before 24 weeks from treatment advice (n, %)	94 (86)
No. of eyes who received intravitreal injections for DME at or after 24 weeks from treatment advice (n, %)	15 (14)

Abbreviations: DM – diabetes mellitus; HBA1C - glycosylated hemoglobin; NPDR – non-proliferative diabetic retinopathy; PDR – proliferative diabetic retinopathy; ETDRS – Early Treatment Diabetic Retinopathy Study; DME – diabetic macular edema

Table 2 compares the demographic, clinical, and OCT findings between the two groups of patients with CI-DME when intravitreal therapy was recommended. The average time between recommending treatment and receiving treatment in Group 1 (early) was 2.237 ± 3.607 weeks and 31.24 ± 7.456 weeks in Group 2 (late).

**Table 2:** Demographic, clinical and optical coherence tomography (OCT) finding comparisons at the time of treatment advice between the two study groups:

	Early treatment group	Delayed treatment group	P value
No of eyes (n)	94	15	
No. of patients (n)	81	12	
Males: Females (n)	63:18	9:3	> 0.999
Age (years)	65.93 ± 9.134	62.20 ± 9.536	0.148
Duration of DM (years)	16.99 ± 8.29	15.40 ± 5.06	0.795
HbA1c (%)	8.453 ± 1.03	8.213 ± 0.621	0.565
Moderate NPDR (n, %)	18 (20)	8 (53)	0.008
Severe NPDR (n, %)	38 (40)	0 (0)	0.001
PDR (n, %)	38 (40)	7 (47)	0.779
Visual acuity (ETDRS letters)	50.05 ± 12.1	58.23 ± 11.1	0.314
Central subfield thickness (microns)	527.2 ± 143.7	445.3 ± 107.8	0.036
Average time interval between recommending treatment and receiving treatment (weeks)	2.237 ± 3.607	31.24 ± 7.456	

Abbreviations: DM – diabetes mellitus; HBA1C - glycosylated hemoglobin; NPDR – non-proliferative diabetic retinopathy; PDR – proliferative diabetic retinopathy; ETDRS – Early Treatment Diabetic Retinopathy Study; DME – diabetic macular edema

The demographic profile, duration of diabetes mellitus and glycaemic control between the two groups were comparable. There was no statistical significance between the two groups in terms of VA (p = 0.314) at the time of treatment advice. However, at this time point in the study, the CSFT in group 1 eyes was significantly higher than in group 2 eyes (p = 0.036).

Table 3 compares the clinical grading of DR, VA, and OCT measurements between the two groups at the time of treatment and one year after starting the therapy. Between the two groups, eyes in Group 2 had significantly better VA and lower CSFT than eyes in Group (1) However, at the 1-year follow-up time point after starting treatment, Group 2’s VA (53.4 ± 12.67) was significantly lower than Group 1’s (57.38 ± 20.01). At the 1-year follow-up visit, the CSFT in Group 1 decreased while it increased in Group (2) After one year of treatment, there was an average improvement of + 7.6 ETDRS letters in Group 1 and a decline of -6.9 ETDRS letters in Group 2.

**Table 3:** Demographic, clinical and optical coherence tomography (OCT) finding comparisons between the two study groups at the time of injection and at 1 year post commencement of treatment:

	Early treatment group (n = 94)	Delayed treatment group (n = 15)	P value
Worsening in DR severity at least by 1 step at the end of 1 year (n, %)	13 (14)	4 (27)	0.247
Mean VA (ETDRS letters) at the time of injection	49.74 ± 16.3	56.27 ± 15.2	0.003
Mean CSFT at the time of injection	527.2 ± 143.7	462.5 ± 73.57	< 0.001
Mean VA (ETDRS letters) 1-year after treatment	57.38 ± 20.01	51.4 ± 12.67	0.170
Mean change in VA (ETDRS letters) at the end of 1 year	7.638 ± 17.96	-6.867 ± 11.65	0.001
Mean CSFT 1-year after treatment	422.6 ± 158.9	477.8 ± 92.94	0.846

Abbreviations: DR – diabetic retinopathy; VA – visual acuity; CSFT – central subfield thickness; ETDRS – Early Treatment Diabetic Retinopathy Study

Table 4 compares changes in VA and CSFT between groups at the one-year follow-up visit. At the 1-year follow-up visit after starting the therapy, there was a significant improvement in VA and a reduction in CSFT in group 1 and vice versa in group 2 (p < 0.05).

**Table 4:** Changes in visual acuity and CSFT in eyes following treatment in the prompt and delayed treatment groups at the 1 year follow up visit:

		Early treatment group (n = 94)	Delayed treatment group (n = 15)	P value^#^
VA (ETDRS letters)	At the time of injection	49.74 ± 16.3	60.27 ± 15.2	0.003
	After 1 year post treatment	57.38 ± 20.01	53.4 ± 12.67	0.170
	P value^*^	< 0.001	0.042	
CSFT	At the time of injection	527.2 ± 143.7	462.5 ± 73.57	< 0.001
	After 1 year post treatment	422.6 ± 158.9	477.8 ± 92.94	0.846
	P value^*^	< 0.001	0.021	

Abbreviations: VA – visual acuity; CSFT – central subfield thickness; ETDRS – Early Treatment Diabetic Retinopathy Study; P^#^ - P value calculated between early and delayed treatment groups; P^*^ - P value calculated at the time of injection and after 1 year-post treatment

When compared to eyes where treatment was started early, eyes where treatment was delayed required a greater number of intravitreal anti-VEGF and steroid injections and focal/grid thermal laser photocoagulation sessions. This is noted in Table 5.

**Table 5:** Comparisons in the treatments received during the 1-year study period between both groups:

	Early treatment group (n = 94)	Delayed treatment group (n = 15)	P value
Median number of Anti-VEGF injections taken during 1 year (25 – 75% percentile interquartile range)	1 (1–3)	3 (2–4)	0.182
Median no of intraocular steroid injections taken during 1 year (25 – 75% percentile interquartile range)	1 (0-1.25)	4 (2–4)	< 0.001
No. of eyes treated with focal/grid thermal laser photocoagulation	12 (13)	8 (53)	0.001
Median no. of focal/grid thermal laser photocoagulation sessions	1 (0-1.25)	4 (2–4)	< 0.001

Abbreviations: VEGF – vascular endothelial growth factor

### Patients with ≥ 5 ETDRS letter loss

The current study found that 18 of the 94 (19%) eyes in the early treatment group and 8 of the 15 (53%) eyes in the deferred treatment group lost ≥ 5 ETDRS letters at year 1. This was significantly higher in the delayed treatment group than in the early treatment group (p = 0.008). In the early treatment group, the most common causes of vision loss were worsening macular edema in 10 (56%) eyes, cataract development in 6 (33%) eyes, and ELM-EZ discontinuity in 2 (11%) eyes. Vision loss in the delayed treatment group was primarily attributed to ELM-EZ discontinuity in 5 (62%) eyes, worsening macular edema in 2 (25%) eyes, and cataract development in one (13%) eye.

### Summarizing the characteristics of the delayed treatment group

This group included 15 eyes of 12 patients who had CI-DME treatment 24 weeks after receiving treatment advice. This group consisted of 9 (75%) males and 3 (25%) females. The average age of the patients in this group was 62.20 ± 9.536 years, and the average duration of diabetes was 15.40 ± 5.06 years. The average time it took patients to receive intravitreal injections after receiving treatment advice was 31.24 ± 7.456 weeks. Patients cited the following reasons for not considering intravitreal therapy at the time of treatment recommendation: (a) absence of visual symptoms and good presenting VA in eight of twelve (66%) patients, (b) inability to achieve optimal sugar levels for intravitreal injection in two (17%) cases, and (c) high treatment costs in two (17%) additional cases. Between the different time points (i.e., at the time of treatment advice, treatment injection and 1-year follow up) in the study, the delayed treatment group showed progressive VA worsening and CSFT thickening. More than 50% of the eyes in the delayed treatment group showed a drop of ≥ 5 ETDRS letters in the study. The primary reason for the reduction of VA was the worsening of DME. In the study, the delayed treatment group required a greater median number of anti-VEGF and steroid intravitreal injections. The type of anti-VEGF agent used did not differ between the two groups. During the one-year follow-up, patients in the delayed treatment group required more focal thermal laser treatment sessions.

## Discussion

This study described the treatment outcomes of a small but extremely important group of patients who received delayed treatment for DME for the first time in a real-world clinical setting. Over a one-year follow-up period after starting treatment, eyes with delayed therapy required significantly more intravitreal injections, had worsening of the central retinal thickness, and poor VA improvements when compared to eyes with early disease treatment. Furthermore, in the delayed treatment group, the proportion of patients who lost 5 ETDRS letters or more was higher than in the early treatment group.

According to the findings of this study, approximately 14% of the patients who needed DME treatment failed to adhere to the management protocol. There are a variety of reasons for denying treatment by the patient, including mild-moderate vision loss, fear of repeated injections and complications associated with the procedure, inability to come for regular, timely follow-up visits, deranged glucose level, and, most importantly, treatment costs [16, 17]. At the time of treatment advice, the VA and CSFT in the delayed treatment group were significantly better than those in the early treatment group in this study. The delayed treatment group’s VA had decreased further by the time they received treatment. Thus, the absence of significant vision loss during the initial disease course and further worsening over time could be one of the primary reasons for patients to postpone early treatment and seek treatment later.

Fluid accumulation in DME is caused by hyperpermeable deep retinal capillaries as well as poor resorption by the RPE at the fovea. This causes fluid to accumulate within the intraretinal layers and beneath the fovea in DME [18]. There is a weak relationship between OCT-measured CSFT and VA [19]. Short-term changes in the CSFT cannot be used as surrogate markers to predict long-term changes in VA. Several studies have clearly demonstrated that the continuity and integrity of the outer retinal layers, namely the ELM, EZ, and RPE, determine long-term VA changes. Chronic untreated or persistent DME is linked to discontinuity and damage to the outer retinal layers, resulting in permanent vision loss [20–22]. In the current study, we observed that patients in the delayed treatment group had poor VA improvement over a 1-year follow-up period after initiation of therapy. Most patients in the delayed treatment group with reduced VA showed discontinuity of the ELM-EZ layers (n = 5, 62%). This confirms that persistent edema over a long period is responsible for outer retinal layer changes at the fovea and poor VA. Thus, early treatment of DME with intensive therapy prior to the development of changes in the outer retinal layers may benefit long-term VA. A study by Angermann et al. emphasized the significance of adherence to treatment protocol in DME patients [17]. They discovered that patients with good therapy adherence had better visual outcomes and a lower risk of disease progression than patients with poor therapy adherence. Furthermore, visual outcomes were poor in DME cases that were lost to follow-up over a 48-week follow-up period. This study highlights the importance of early therapy in DME cases, as well as good adherence to treatment, to maximize visual benefits.

In the treatment of DME, focal/grid laser photocoagulation, VEGF inhibitors and corticosteroids have remained the mainstay. In the current study, we discovered that patients in the early treatment group required fewer intravitreal steroid injections over a one-year period. In contrast, the delayed treatment group required a greater proportion of intravitreal anti-VEGF agents and steroids and focal laser treatment sessions than the early treatment group. The DRCR.net Protocol K evaluated the outcomes of patients with treatment-naive CI-DME who were treated with focal/grid laser photocoagulation [23]. According to the study, a significant number of eyes did not show a reduction in CSFT of more than 10% at the 16-week follow-up visit. As a result, the limited role of focal/grid laser photocoagulation in CI-DME cases is highlighted. In the management of DME, poor response or resistance to routine anti-VEGF agents is a reality. A recent study by Elnahry et al. found that OCT and OCT-angiography markers, such as a higher CSFT and a smaller foveal avascular zone area, as well as increased vessel densities in superficial parafovea and deep fovea, were associated with a better response to monthly intravitreal bevacizumab injections than in non-responders [24]. Patients who do not respond adequately to standard anti-VEGF medications may benefit from treatment with new and potent anti-VEGF molecules such as brolucizumab and faricimab [25, 26]. Long-term, chronic, untreated DME appears to develop resistance and a poor treatment response to routine antiangiogenetic drugs in general, according to studies [27, 28]. These eyes can also be treated with potent anti-VEGF molecules like faricimab [26]. Corticosteroid therapy, which controls the crucial role of inflammation in DME, is used to treat persistent, chronic DME [29]. Increased intraocular corticosteroid use puts patients at risk for intraocular hypertension and cataract formation [29]. Therefore, early treatment with a VEGF inhibitor appears to be the optimal method for treating DME. In our study, the delayed untreated group could have had a higher proportion of anti-angiogenic therapy-resistant cases. These eyes were treated with intravitreal corticosteroid therapy as opposed to the standard anti-VEGF treatment. For the treatment of resistant cases, newer and more potent anti-VEGF molecules, such as brolucizumab and faricimab, were not available at the time the study was conducted. This emphasizes the fact that early treatment of DME with routinely available anti-VEGF agents such as bevacizumab, ranibizumab, and aflibercept may result in a favourable response and eliminate the need for costly and less frequently available drugs such as brolucizumab and faricimab. Additionally, complications caused by intraocular corticosteroid use could be avoided.

In comparison to other macular pathologies that require frequent intravitreal injections, such as neovascular age-related macular degeneration, compliance and adherence to the DME treatment protocol was poor, which affects the final visual outcome [30]. As a result, strategies to bind DME patients to regular intravitreal injections must be identified from the start, based on the causes of poor compliance and adherence, particularly the social determinants. These may include strategies such as raising awareness about the disease and the importance of early intervention, policies to increase the number of patients covered by medical insurance, forcing insurance companies to cover treatment with biosimilar anti-VEGF agents, more widespread use of less expensive biosimilar anti-VEGF agents, and reducing hospital visits by providing home vision monitoring devices and home-based OCT scans.

This study has several limitations. To begin, the sample size of the delayed treatment group was smaller. The disproportionate number of cases between the two groups was caused by the strict inclusion criteria. Second, the initial visit itself lacked information regarding the duration of DME. Patients in the early treatment group who exhibited a suboptimal response to therapy may have had a longer duration of DME. Third, the treatment of DME lacked a step-by-step approach, particularly in the delayed treatment group. The treatment was entirely at the discretion of the retina specialist who administered it. Anti-VEGF therapy as the initial treatment, followed by corticosteroids and/or focal laser in non-responsive cases, could have provided additional evidence that chronic DME cases are poor anti-VEGF drug responders. Other factors, such as social and economic health determinants, were not considered in this research. Fifth, a questionnaire could have helped determine the reasons for DME patients’ poor compliance and adherence to therapy. Future plans should include a multicentric study with a larger sample size, well-defined treatment strategies, and a focus on identifying the causes of poor compliance and adherence to intravitreal drug therapy. To improve visual outcomes in DME eyes, efforts should be focused on decreasing the proportion of patients who receive delayed treatment.

Finally, it can be concluded that a collaborative effort should be made by clinicians, pharmaceutical and insurance companies, as well as the hospital administrative team, to encourage and motivate patients to begin intensive therapy early in the disease course in order to reduce the burden of a greater number of intravitreal injections and VA loss over time.

## Data Availability

The datasets used and/or analysed during the current study are available from the corresponding author on reasonable request.

## Declarations

### Animal research

“This article does not contain any studies with animals performed by any of the authors.”

## Abbreviations

DR: diabetic retinopathy
DME: diabetic macular edema
VEGF: vascular endothelial growth factor
CI-DME: center-involving diabetic macular edema
VA: visual acuity
DRCR.net: Diabetic retinopathy clinical research network
CSFT: central subfield thickness
OCT: optical coherence tomography
ELM: external limiting membrane
EZ: ellipsoid zone
RPE: retinal pigment epithelium
ETDRS: Early Treatment Diabetic Retinopathy Study
